# Risk of early death after treatment with curative intent for head and neck squamous cell carcinoma: a retrospective population-based study

**DOI:** 10.2340/1651-226X.2025.42202

**Published:** 2025-03-04

**Authors:** Mahmoud Bazina, Rayan Nikkilä, Aaro Haapaniemi, Leif Bäck, Sami Ventelä, Antti Mäkitie

**Affiliations:** a Department of Otorhinolaryngology – Head and Neck Surgery, University of Helsinki and HUS Helsinki University Hospital, Helsinki, Finland; b Research Program in Systems Oncology, Faculty of Medicine, University of Helsinki, Helsinki, Finland; c Finnish Cancer Registry, Institute for Statistical and Epidemiological Cancer and Research, Helsinki, Finland; d Department of Oral and Maxillofacial Surgery, Lahti Central Hospital, Päijät-Häme Joint Authority for Health and Wellbeing, Lahti, Finland; e FICAN West Cancer Centre, Turku, Finland; f Department for Otorhinolaryngology – Head and Neck Surgery, University of Turku and Turku University Hospital, Turku, Finland; g Turku Bioscience Centre, University of Turku and Åbo Akademi University, Turku, Finland; i Division of Ear, Nose and Throat Diseases, Department of Clinical Sciences, Intervention and Technology, Karolinska Institutet and Karolinska Hospital, Stockholm, Sweden

**Keywords:** Mortality, risk factors, curative intent, surgery, radiotherapy, chemoradiotherapy

## Abstract

**Background and purpose:**

Knowledge regarding the risk factors for early death in patients with head and neck squamous cell carcinoma (HNSCC) is scarce. This study aims to evaluate the rate of early death (during or within 6 months of treatment) and its associated risk factors in HNSCC patients treated with curative intent.

**Materials and methods:**

A retrospective, population-based analysis of all HNSCC patients (*n* = 762) treated with curative intent at the Helsinki University Hospital (Helsinki, Finland) during 2012–2015 was conducted. Using the chi-square test, associations between categorical variables were assessed. Univariate and multivariate analyses were performed to identify independent factors for early death.

**Results:**

The rate of early death was 10.1% with a median age of 70 years at diagnosis. Advanced stage, smoking > 40 pack-years, and heavy alcohol consumption were associated with increased odds of early death. Elevated thrombocyte levels > 380 (× 10⁹L) were observed more frequently in the early-death group when comparing the levels with the late-death group (*p* < 0.01). However, only age (odds ratio [OR] 1.05; 95% confidence interval [CI]:1.02–1.08), T4 class (OR 5.98; 95% CI: 2.60–13.74), N2 class (OR 2.98; 95% CI: 2.60–13.74), and N3 class (OR 12.24; 95% CI: 2.99–50.19) emerged as independent risk factors for early death.

**Interpretation:**

Early death risk is increased in older patients and those with advanced-stage HNSCC. Elevated thrombocyte count requires further studies to assess its utility as a potential clinical marker.

## Introduction

According to GLOBOCAN estimates in 2022, head and neck cancer (HNC) accounted for an estimated 947,000 new cases and 482,000 deaths worldwide [1]. Approximately 90% of HNCs are head and neck squamous cell carcinomas (HNSCCs) [2]. While treatment with curative intent aims at long-term survival, HNC is also associated with early death within 6 months of diagnosis or after treatment with rates ranging from 3% to 11% [3–6]. Most recently, in Sweden, Talani et al. [3] reported that 6-month death rate after HNC diagnosis among patients treated with curative intent decreased from 4.7% (2008–2012) to 2.5% (2017–2020).

The research on the risk of early death among HNC patients remains, however, scarce with most the existing studies focusing on a narrow range of clinical factors, such as tumor stage and location, and treatment methods. Previously published studies have shown that older patients with advanced disease, high WHO score, primary tumor in the hypopharynx, and those receiving palliative treatment are more likely than others to die of HNC within 6 months of diagnosis [4, 7, 8]. n addition, patients with HPV-positive (Human papillomavirus) oropharynx cancer commonly have better survival than patients with HPV-negative tumors, and smoking is a negative prognostic factor for overall survival in patients with HPV-positive cancer [9]. Indeed, a notable lack of information exists regarding lifestyle factors, such as alcohol use and smoking habits, HPV status, patient comorbidities, and postoperative complications.

The current study aims to identify risk factors associated with early death among patients with HNSCC undergoing treatment with curative intent. Identifying these factors may aid in optimizing treatment strategies and healthcare resources. Our tertiary care center is the biggest healthcare provider in Finland (5.6M) and is responsible for delivering treatment for 2.2 million residents, allowing us to conduct a population-based study.

## Materials and methods

This retrospective cohort comprises all patients diagnosed with a histologically confirmed HNSCC and treated with curative intent at the Helsinki University Hospital (HUS, Helsinki, Finland) between January 1, 2012, and December 31, 2015. The data were gathered in collaboration with the HUS Data Service. Patients were identified by ICD-10 diagnosis codes (International Classification of Diseases 10th revision). The included tumor sites were: lip (C00.0–2, C00.6, C00.8, C00.9), oral cavity (C00.3, C00.4, C02, C03, C04, C05, C06), oropharynx (C01.9, C05.1, C05.2, C05.8, C05.9, C09, C10), salivary glands (C07, C08), nasopharynx (C11), hypopharynx (C12, C13), larynx (C10.1, C32), nose and paranasal sinuses (C30.0, C31), and HNSCC unknown primary (C77.0).

Patients’ hospital records were reviewed and clinicopathological data were collected on the following: sex, age, smoking history and alcohol consumption habits before diagnosis, date of diagnosis according to the pathology report, tumor location, tumor class, node class, and distant metastasis class (i.i.e. Union for International Cancer Control UICC TNM classification, 7th edition), p16 status, histological grade, multidisciplinary tumor board decision and intent of treatment (curative intent included only), surgery, postoperative or definitive oncological treatment – radiotherapy (RT) or chemoradiotherapy (CRT). Outcome and follow-up data comprised residual tumor growth and its treatment, location and date of disease recurrence, date and status at last follow-up, and date and cause of death.

Moreover, patients who died within 2 years of treatment were identified and additional data were collected: Age-adjusted Charlson Comorbidity Index (ACCI) and Adult Comorbidity Evaluation-27 (ACE-27) as indicators of comorbidity, Clavien-Dindo classification system to assess surgical complications and preoperative thrombocyte count. Hemoglobin and albumin levels, if available, were recorded for patients in the early-death group.

Patients were excluded if they had received prior cancer treatment to the head and neck or were treated with palliative intent. Patients diagnosed with a synchronous second primary malignancy were not excluded from the study.

Descriptive statistics were calculated for numerical and categorical variables (Table 1). Early death was defined as death during treatment or within 6 months of the end of treatment. This ensures that both the treatment period and the immediate post-treatment period are included in the analysis of early deaths, allowing for a more comprehensive assessment of survival. Late death was defined as death within 7–24 months of the end of treatment. These patients were compared with patients alive at 2 years. The association between categorical variables was tested using the chi-square test. Means were compared using *t*-tests. Survival analysis after treatment completion was conducted using the Kaplan–Meier method and differences between survival curves were assessed with the log-rank test. Unadjusted odds ratios (ORs) were calculated by conditional maximum likelihood estimation and 95% confidence intervals (CI) using exact methods. A generalized linear model was employed to calculate adjusted ORs. ORs were adjusted for sex, age, smoking history, alcohol use, T class, N class, and treatment. The variables were selected a priori. A *p*-value less than 0.05 was regarded as having statistical significance. All statistical analyses were performed using R software (The R Project for Statistical Computing, version 4.3.1).

**Table 1:** Characteristics and survival of patients: Chi-squared test for patients dead within 6 months and 7–24 months of treatment compared to patients alive at 2 years.

Variables	Overall population		Died during or within 6 months of treatment (Early death)		Died within 7–24 months of treatment (Late death)		Alive at 2 years after treatment	
	*N*	%	*N*	%	*N*	%	*N*	%
	718		77	10.1	97	12.7	544	71.4
**Median age at diagnosis Mean (range)**			70 68.6 (33–86)		67 67.1 (31–100)		64 63.6 (17–93)	
**Age range (years)**								
18–39	16	2	1	1	3	3	12	2
40–59	177	25	10	13	21	22	146	27
60–79	453	63	54	70	59	61	340	63
80+	72	10	12	16	14	14	46	8
**Sex**								
Male	500	70	56	73	67	69	377	69
Female	218	30	21	27	30	31	167	31
**Smoking history**								
<10 pack-years (including non-smokers)	203	28	15	19	20	21	168	31
10–20 pack-years	38	5	4	5	2	2	32	6
20–30 pack-years	50	7	4	5	10	10	36	7
30–40 pack-years	79	11	8	10	9	9	62	11
≥40 pack-years	320	45	42	55	48	49	230	42
No information	28	4	4	5	8	8	16	3
**Alcohol use**								
<10 drinks/week (including non-drinkers)	508	71	47	61	63	65	398	73
10–20 drinks/week	45	6	3	4	9	9	33	6
>20 drinks/week	132	18	21	27	18	19	93	17
No information	33	5	6	8	7	7	20	4
**Tumor location**								
Lip	17	2	0	0	2	2	15	3
Oral cavity	320	45	38	49	44	45	238	44
Oropharynx	174	24	16	21	15	15	143	26
Hypopharynx	25	3	3	4	11	11	11	2
Nasopharynx	8	1	0	0	2	2.0	6	1
Larynx	130	18	13	17	14	14	103	19
Nose and paranasal sinus	23	3	4	5	4	4	15	3
Salivary gland	4	1	1	1	1	1	2	0
Unknown primary carcinoma	16	2	2	3	4	4	10	2
**T class**								
T0	17	2	2	3	5	5	10	2
T1	284	40	10	13	17	18	257	47
T2	166	23	15	20	27	28	124	23
T3	73	10	11	14	13	13	49	9
T4	177	25	38	50	35	36	104	19
**N class**								
N0	390	54	22	29	39	40	329	60
N1	79	11	11	14	14	14	54	10
N2	228	32	36	47	36	37	156	29
N3	20	3	7	9	8	8	5	1
**M class**								
M0	713	99.5	75	99	95	98	543	100
M1	4	0.5	1	1	2	2	1	0
**Stage**								
I	222	31	7	9	6	7	209	38
II	192	27	7	9	49	53	136	25
III	126	18	10	13	36	39	80	15
IV	172	24	52	68	1	1	119	22
**Histological grade**								
Grade 1	87	12	7	9	6	6	74	14
Grade 2	301	42	38	49	50	52	213	39
Grade 3	284	10	30	39	36	37	218	40
No information	46	6	2	3	5	5	39	7
**p16 status for oropharyngeal tumors**								
Negative	31	18	8	50	8	53	15	10
Positive	133	76	8	50	6	40	119	83
No information	10	6	0	0	1	7	9	6
**Treatment modality**								
Only surgery	236	33	14	18	22	23	200	37
Surgery and RT	149	21	22	29	22	23	105	19
Surgery and CRT	103	14	18	23	16	16	69	13
RT	68	9	8	10	12	12	48	9
CRT	162	23	15	19	25	26	122	22
**Disease recurrence after treatment**								
**No**	566	79	52	68	43	44	471	87
**Yes**	152	21	25	32	54	56	73	13

RT: radiotherapy; CRT: chemoradiotherapy.

Study permission was granted by the Research Administration of the Helsinki and Uusimaa Hospital District (HUS/307/2019). Research Ethics Board approval for the retrospective registry study was not needed under Finnish legislation due to the retrospective chart review design of the study.

## Results

**Figure 1:**
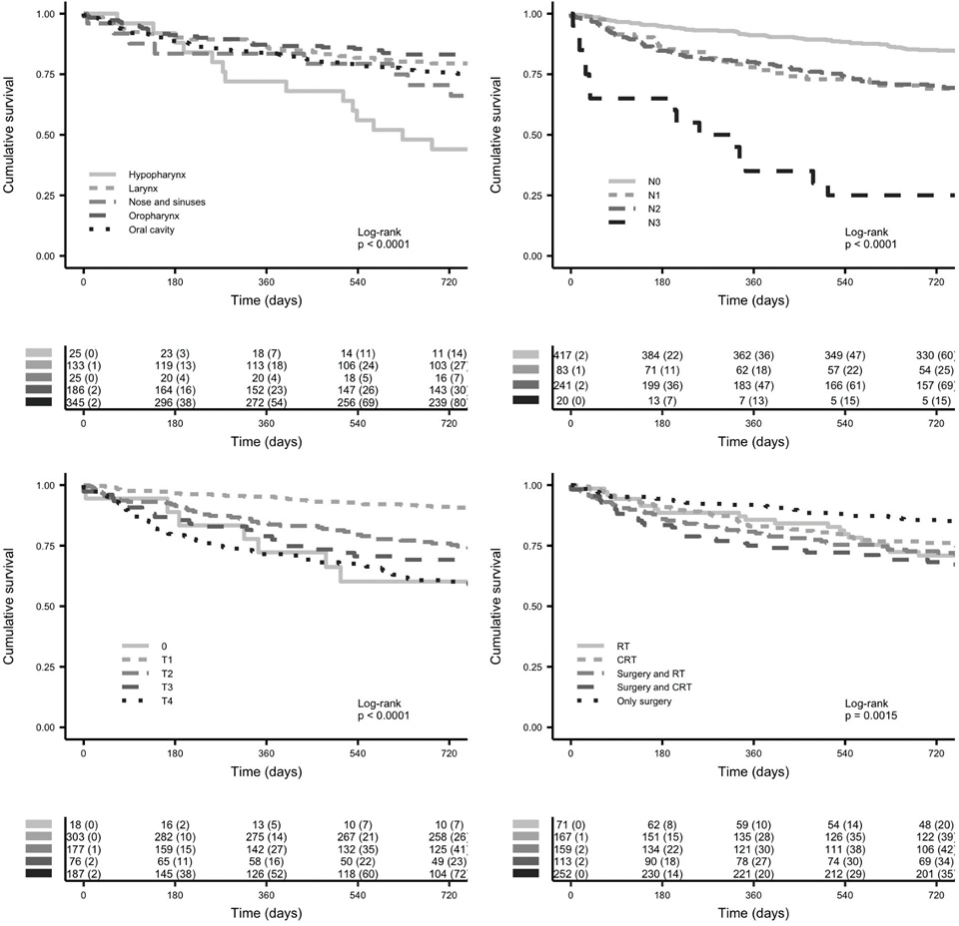
Overall survival for 762 patients treated with curative intent at the HUS Helsinki University Hospital (Helsinki, Finland) between January 1, 2012 and December 31, 2015, stratified by tumor location, tumor class, node class, and treatment.

The characteristics of the study population are given in Tables 1 and 2. Of the 762 patients, 77 (10.1%) died within 180 days of treatment, 97 (12.7%) died between 181 and 731 days, and 544 patients (71.4%) were alive at 2 years. A total of 44 patients (5.6%) were lost to follow-up within 2 years after treatment completion: 20 (2.6%) within 180 days, and 24 (3.1%) within 181–731 days. We divided the risk factors for early death into three categories: patient-, tumor-, and treatment-related factors. Figure 1 shows the overall survival for the 762 patients stratified by tumor location, tumor class, node class, and treatment.

**Table 2:** Patients’ characteristics and survival. Chi-squared test for patients dead within 6 months compared to patients dead within 7–24 months.

Variables	Died during or within 6 months of treatment (Early death)		Died within 7–24 months of treatment (Late death)		*p*
	*N*	%	*N*	%	
**ACE-27 score**					n.s.
1	40	52	58	60	
2	18	23	18	19	
3	19	25	21	22	
**ACCI score**					n.s.
0	2	3	4	4	
1	6	8	14	14	
2	13	17	16	16	
3	15	19	20	21	
4	12	16	14	14	
≥ 5	29	38	29	30	
**Preoperative thrombocyte count (X10^9^L)**					**< 0.01**
< 150	6	8	13	13	
150–380	53	69	77	79	
> 380	18	23	7	7	
**Clavien-Dindo Classification**					n.s.
1	23	30	32	33	
2	15	19	17	18	
**3–4**	15	19	17	18	
5	2	3	0	0	
No surgery	22	29	31	32	
**Cause of death**					**< 0.05**
HNSCC/treatment-related	58	75	56	58	
Other	13	17	18	19	
Unknown	6	8	23	24	

Significant *p*-values (< 0.05) are shown with bolded text.

*n.s.:* non-significant *p*-value; HNSCC: head and neck squamous cell carcinoma; ACCI: Age-adjusted Charlson Comorbidity Index; ACE-27: Adult Comorbidity Evaluation-27.

### Patient-related factors

#### Sex

Most of the patients were male (*n* = 500, 78%), with a 11% (*n* = 56) death rate during or within 6 months after treatment. For female patients, 10% (*n* = 21) died within 6 months. Conversely, 75% (*n* = 377) of all men and 77% (*n* = 167) of all women were alive at 2 years.

#### Age

Patients in the early-death group and patients alive at 2 years had a median age of 70 years (mean 68.6, range 33–86) and 64 years (mean 63.6, range 17–93), respectively. Each additional year of age induced the odds of early death by a factor of 5% (OR 1.05, 95%-CI 1.02–1.08).

#### ACE-27 score

In the early-death group, 40 (52%) patients had an ACE-27 score of 1, 18 (23%) patients had a score of 2, and 19 (25%) patients had a score of 3. Correspondingly, in the late-death group, 58 (60%) patients had an ACE-27 score of 1, 18 (19%) patients had a score of 2, and 21 (22%) patients had a score of 3 (Table 2). There were no statistically significant differences between early- and late-death groups regarding the ACE-27 grading.

#### ACCI-score

The early- and late-death groups included 29 (38%) and 29 (30%) patients with an ACCI score of ≥5, respectively (*p* = n.s., Table 2).

#### Smoking and alcohol history

Patients with a history of 20 alcoholic drinks per week had higher odds of early death than patients consuming less than 10 alcoholic drinks a week (OR 1.83, 95% CI: 1.03–3.15). Similarly, patients with a smoking history of more than 40 pack-years had a higher OR (1.91) than individuals with less than 10 pack-years (1.0). However, these variables were not statistically significant in the multivariate analysis.

#### Preoperative thrombocyte count, hemoglobin, and albumin

Differences were observed in the thrombocyte count when comparing early- and late-death groups (*p* = 0.008). Twenty-three per cent of early deaths and 7% of late deaths had a thrombocyte count > 380 (× 10⁹L) (*p* < 0.01). A subset analysis of tumor stage showed that 56% of patients with early death and 25% of those with late death had a thrombocyte count > 380 (× 10⁹L) along with N2 class.

In the early-death group, hemoglobin levels were available for 95% of the patients. The median hemoglobin level was 130 g/L (range, 82–177) for males and 123 g/L (range, 95–143) for females. Preoperative serum albumin levels were available for 57% of the patients, with a median level of 33 g/L (range, 18–42). Postoperative albumin levels were available for 65% of the group, with a median of 29 g/L (range, 17–42).

### Tumor-related factors

#### Site

The majority of HNSCCs occurred in the oral cavity, accounting for 49% (*n* = 38), 45% (*n* = 44), and 44% (*n* = 238) of tumors in the groups with early death, late death, and those alive at 2 years, respectively. Hypopharyngeal cancer represented 4% (*n* = 3), 11% (*n* = 11), and 2% (*n* = 11) of the tumors in the groups with early death, late death, and those alive at 2 years, respectively.

#### Tumor class

Advanced T class correlated with a worse survival prognosis. Patients with a T3 class had an OR of 4.74 (95% CI: 1.90–11.97) and patients with a T4 class had an OR of 7.33 (95% CI: 3.67–16.03) for early death when compared with patients with a T1 tumor. Adjusted OR for T3 tumors was 4.10 (95% CI: 1.44–11.72; *p* = n.s) and for T4 tumors 5.98 (95% CI: 2.60–13.74; *p* < 0.001) (Table 3).

**Table 3:** Univariate and multivariate analysis. Odds ratios (ORs) with 95% confidence intervals for death within 6 months. Adjusted ORs for sex, age, smoking history, alcohol use, T class, N class, and treatment.

Variable	Died during or within 6 months of treatment	Alive over 6 months after treatment	OR	95% CI	*p*	Adjusted OR *	95% CI	*p*
**No. of patients**	77	665						
**Age**						1.05	1.02–1.08	**< 0.01**
**Sex**								
Male	56	459	1			1		
Female	21	206	1.19	0.71–2.06	n.s.	0.68	0.36–1.27	n.s.
**Smoking history**								
< 10 pack-years	15	197	1			1		
10–30 pack-years	8	84	1.26	0.49–3.04	n.s.	2.25	0.82–6.20	n.s.
30–40 pack-years	8	72	1.47	0.56–3.57	n.s.	1.46	0.51–4.24	n.s.
> 40 pack-years	42	287	1.91	1.05–3.65	**<0.05**	2.09	0.99–4.39	n.s.
**Alcohol use**								
< 10 drinks/week	47	478	1			1		
10–20 drinks/week	3	42	0.76	0.17–2.21	n.s.	0.69	0.19–2.53	n.s.
> 20 drinks/week	21	117	1.83	1.03–3.15	**< 0.05**	1.63	0.84–3.18	n.s.
**Tumor site**								
Oral cavity	38	295	1			1		
Hypopharynx	3	22	1.10	0.24–3.41	n.s.	0.52	0.12–2.34	n.s.
Larynx	13	119	0.85	0.42–1.63	n.s.	1.04	0.43–2.54	n.s.
Nose and paranasal sinuses	4	20	1.59	0.43–4.53	n.s.	1.21	0.30–5.00	n.s.
Oropharynx	16	164	0.76	0.40–1.39	n.s.	0.69	0.30–1.62	n.s.
**T class**								
T1	10	282	1			1		
T2	15	158	2.66	1.17–6.30	**< 0.01**	2.36	0.94–5.97	n.s.
T3	11	65	4.74	1.90–11.97	**< 0.001**	4.10	1.44–11.72	n.s.
T4	38	144	7.33	3.67–16.03	**< 0.001**	5.98	2.60–13.74	**< 0.001**
**N class**								
N0	22	368	1			1		
N1	11	68	2.75	1.22–5.84	**< 0.01**	2.35	0.98–5.69	n.s.
N2	36	192	3.13	1.81–5.56	**< 0.001**	2.98	1.42–6.24	**< 0.01**
N3	7	13	9.33	3.18–25.61	**< 0.001**	12.24	2.99–50.19	**< 0.001**
**Histological grade**								
Grade 1	7	80	1			1		
Grade 2	38	263	1.70	0.77–4.32	n.s.	0.91	0.35–2.37	n.s.
Grade 3	30	254	1.37	0.61–3.54	n.s.	0.61	0.21–1.74	n.s.
**Treatment**								
Surgery	14	230	1			1		
RT	8	62	2.13	0.81–5.26	n.s.	1.52	0.49–4.68	n.s.
CRT	15	150	1.64	0.76–3.55	n.s	0.59	0.20–1.71	n.s.
Surgery and RT	22	134	2.68	1.33–5.56	**< 0.01**	1.33	0.55–3.27	n.s.
Surgery and CRT	18	89	3.30	1.57–7.07	**< 0.001**	1.17	0.39–3.51	n.s.
**Neck dissection**								
No	28	319	1			1		
Yes	49	346	1.61	0.99–2.66	n.s.	0.76	0.31–1.87	n.s.
**Disease recurrence**								
No	52	533	1			1		
Yes	25	132	1.94	1.15–3.23	**< 0.05**	1.42	0.77–2.60	n.s.

CI: confidence interval; OR: odds ratio; RT: radiotherapy; CRT: chemoradiotherapy.

Significant *p*-values (< 0.05) are shown with bolded text.

n.s.: non-significant p-value.

#### Node class

A lower N class correlated with a better prognosis. Patients with an N2 class had an OR of 3.13 (95% CI: 1.81–5.56) and patients with an N3 class had an OR of 9.33 (95% CI: 3.18–25.61) for early death when compared with patients with an N0 class. Adjusted OR for patients with N2 was 2.98 (95% CI: 2.60–13.74; *p* < 0.001) and for those with N3 12.24 (95% CI: 2.99–50.19; *p* < 0.001) (Table 3).

#### p16 status for oropharyngeal tumors

Early- and late-death groups comprised 50% and 40% p16-positive tumors respectively, while 83% of the patients alive at 2 years had a p16-positive tumor.

### Treatment-related factors

#### Treatment modality

Among 236 patients treated by primary surgery alone, the rate of early death was 6% (*n* = 14). The primary surgery was combined with postoperative RT in 149 patients with an early death rate of 15% (*n* = 22). Postoperative CRT was administered to 103 patients with an early death rate of 17% (*n* = 18). Additionally, early death rates were 12% among those with definitive RT and 9% with definitive CRT. After neck dissection (*n* = 379), 13% (*n* = 49) of the patients died within 6 months.

#### Surgical complications

We found no statistically significant differences between early and late death groups when using the Clavien-Dindo classification to assess the severity of surgical complications (Table 2). Only two patients had a Grade 5 complication.

#### Disease recurrence

Early- and late-death groups comprised more disease recurrences (*n* = 25, 32%, *p* < 0.001 and *n* = 54, 56%, *p* < 0.001, respectively) when compared with patients alive at 2 years (*n* = 73, 13%). Disease recurrence led to higher odds of early death (OR 1.94, 95% CI: 1.15–2.23).

#### Cause of death

For 75% (*n* = 58) of the patients in the early-death group and 58% (*n* = 56) in the late-death group, the cause of death was HNSCC. In the early-death group, five patients died during treatment, 25 patients had a disease recurrence, and the remaining 28 patients’ cause of death was not available in the hospital records; however, it was captured from the Finnish Electronic Patient Data Repository (Kanta) and was attributed to HNSCC. The specific cause of death was unavailable for 8% and 24% of the patients in the early- and late-death groups, respectively (see Table 2).

## Discussion

We investigated risk factors associated with early death among 762 patients with HNSCC undergoing treatment with only curative intent. In this cohort, 10.1% of the patients died during or within 6 months after treatment. Advanced T and N class, over 40 pack-years of smoking, and heavy alcohol consumption were associated with increased odds of early death. Additionally, elevated thrombocyte levels > 380 (× 10^9^L) were observed in the early-death group. Additionally, only age, along with T4, N2, and N3 class emerged as independent risk factors for early death.

In Denmark, Jensen et al. [10] analyzed data between 2000 and 2017 from the Danish Head and Neck Cancer Group (DAHANCA). The authors reported a 7.1% mortality within 180 days from the first fraction of RT in 11,419 HNC patients treated with curatively intended RT/CRT with or without prior surgery. Similarly, Kouka et al. [7] reviewed data from 8,288 HNC patients treated between 1996 and 2006 in Germany and reported 30-day, 90-day, and 180-day mortality rates of 1.8%, 5.1%, and 9.6%, respectively, following HNC diagnosis. Previous nationwide research conducted on Finnish and Swedish databases revealed that the mortality rate within 6 months after being diagnosed with HNC was 9.5% in Finland between 1953 and 2012, and 9.8% in Sweden between 2008 and 2013 [5]. Hamilton et al. [8] analyzed 5,658 HNSCC patients treated from 1998 to 2014 in Canada. The 90-day risk of death after starting curatively intended RT, with/without concurrent CRT, or up-front surgery, stood at 3.6%. Aligning with our findings, patients older than 75 years at the time of diagnosis with advanced T and N classification had the highest early mortality. Older age was indeed associated with higher odds of early mortality, in line with previous studies [3, 4, 6–8, 10, 11]. However, Chang et al. found no significant differences in age, gender, TNM stage, cancer location, or the Charlson Comorbidity Index (CCI) between those who died within 60 days after completing chemoradiation and those who survived. Our findings indicated that sex did not affect the early death rate of HNSCC patients, corroborating the results of several earlier studies [4, 6, 8, 10, 12–14]. However, other studies have shown male patients have a higher risk of early death [3, 7, 15, 16].

Classification of comorbidities remains important in head and neck oncology when considering treatment-related outcomes. The ACE-27 index, specifically developed for cancer patients, has been validated in HNSCC patients [17–21]. The Age-adjusted Charlson Comorbidity Index (ACCI) incorporates the patient’s age as an adjustment factor in the final CCI, with several studies demonstrating its effectiveness in predicting both short-term and long-term outcomes in various cancers [22–24]. In our study, there was no significant difference between early- and late-death groups when comparing ACE-27 and ACCI scores. However, we did not account for all comorbidities within the cohort, specifically among patients alive at 2 years, which may have affected our results. Nonetheless, earlier studies have shown a relationship between comorbidities and early mortality. Nieminen et al. [6] found that HNC patients with higher ACE-27 scores faced an increased risk of early postoperative death following microvascular free flap surgery. Patients who died within 6 months of surgery had higher CCI, ACCI, and ACE-27 scores compared to surviving patients. Additionally, ACE-27 has been recognized as a significant independent predictor of 90-day mortality [25]. Bøje et al. [26] showed that 36% of HNSCC patients with comorbidities and a higher CCI score had poorer overall survival (OS) rates. Dixon et al. [14] showed that the presence of multiple comorbidities (ACE-27 ≥1) was associated with an increased risk of death either during the treatment or within 90 days after completing radical RT. Kim et al. [27] identified comorbidity (CCI ≥ 2) at the time of diagnosis as an independent predictor of non-cancer mortality after definitive treatment for advanced-stage HNC.

Our results showed that a history of 20 alcoholic drinks per week carried higher odds of early death than 10 alcoholic drinks (including also non-drinkers). Similarly, patients with a smoking history of more than 40 pack-years had 1.91 times the odds of early death than individuals with less than 10 pack-years (including non-smokers). However, these variables were not statistically significant in the multivariate analysis. Hoff et al. [28] showed that smoking during RT negatively impacts HNC treatment outcomes and the risk of death increases with each additional pack-year of smoking. Schlumpf et al. [29] conveyed an early mortality rate of 5.4% in 167 HNSCC patients who died during or within 30 days after completion of concurrent RT/CRT. Further, 90% of the patients who died during treatment had smoked on average 54 pack-years and consumed alcohol. However, none of these factors were significantly associated with death while under treatment. Denissoff et al. [30] reported that continuation of alcohol use, even at a moderate level (10–20 drinks/week) is associated with increased mortality risk after receiving HNSCC diagnosis, independent of age at diagnosis, tumor stage, and tobacco use status. However, drinking behavior around the time of diagnosis of HNC was not associated with a higher mortality risk in the analysis by Beynon et al. [31].

We observed no differences in tumor location between early-death patients and patients alive at 2 years. Yet, Hamilton et al. [8] showed that oral cavity cancer was associated with an increased risk of early mortality when compared with oropharyngeal cancer. This observation is supported by a Surveillance, Epidemiology, and End Results (SEER) population-based analysis, particularly for tumors located in the tongue [32]. Talani et al. [3, 4] identified hypopharyngeal cancer as a significant independent risk factor for early mortality, aligning with prior research [10, 16, 33]. Kouka et al. [7] further highlighted the fact that the sites of the oral cavity (OR 3.47), oropharynx (OR 3.01), and hypopharynx (OR 3.27) were associated with significantly higher 180-day mortality in multivariate analyses.

Our findings indicate that advanced T or N class increase early mortality, with T4, N2, and N3 class independently linked to this risk after curative treatment. This aligns with previous research [6–8]. In addition, Tighe et al. [11] found that extracapsular spread was an independent risk factor for 30-day postoperative mortality for HNSCC. Stage IV disease is an independent risk factor for early death in accordance with previous studies [4, 7, 10].

Previous studies have described an association between elevated thrombocytes and poor survival in HNSCC patients, supportive of our observations. A meta-analysis by Takenaka et al. [34] reported that thrombocyte counts greater than the cutoff value (ranging from 150 to 400 × 10^9^L) were associated with poorer OS (HR 1.81; 95% CI 1.16–2.82). The physiological roles of thrombocytes can facilitate cancer progression by binding immune cells and modulating the immune response. Furthermore, thrombocytes inhibit tumorlytic activity by aggregating around tumor cells and may promote tumor growth and metastasis [34]. Indeed, antithrombotic therapies have been associated with better oncologic outcomes in patients with HNSCC [35, 36]. In this cohort, the T class distribution between early- and late-death groups is relatively similar, which suggests that the association between thrombocyte count and early death is less likely to be confounded by the T class. However, the N class distribution shows more variation between the groups, indicating that nodal metastasis could play a role in this association. Indeed, 56% of early-death patients and 25% of late-death had a thrombocyte count > 380 (× 10⁹L) along with N2 class. Due to the limited number of cases, a multivariate analysis would not be feasible. Giannakeas et al. stated that higher thrombocyte counts were associated with cancer-specific death for various cancer sites. Therefore, thrombocyte count might indicate residual disease post-treatment and could be a potential risk stratification tool to guide the need for intensified treatment [37].

In previous reports, low albumin levels have been associated with early mortality in univariable logistic regression analysis [6, 12]. Also in our series, both the pre- and post-operative median albumin levels in the early-death group were slightly lower than normal values. In HNC patients, a hemoglobin level below 100 g/L at diagnosis has been identified as an independent risk factor of noncancer mortality [27]. Additionally, pre-treatment hemoglobin levels below the normal lower range have been recognized as a significant independent predictor of 90-day mortality [25]. In the present cohort, the median hemoglobin level was 130 g/L for males (slightly lower than normal) and 123 g/L for females (within normal range).

The causes of death were investigated for patients who died within 2 years. In the early-death group, 32% of patients had a recurrence, while 75% died from HNSCC. Some patients in the early-death group died during treatment, which does not count as a recurrence, as these patients did not survive long enough for a recurrence to be documented. Furthermore, the number of deaths from HNSCC includes cases where recurrence data may not have been explicitly recorded but where the cause of death was attributed to the primary disease (28 and 2 such cases for the early and late death groups, respectively). This explains why in the late-death group, 56% of patients had a recurrence but 58% of patients died from HNSCC.

Limitations of our study include those inherent in the retrospective design, including some missing data on smoking and alcohol history especially concurrent use, and specific cause of death. In addition, we were not able to assess the impact of factors such as pre-treatment body weight, nutrition status, low fat-free mass index, or peripheral blood total lymphocyte count, all of which have been shown to confer a higher risk of early mortality [12, 14, 27, 38, 39]. Also, due to the small cohort size, the relationship between thrombocyte counts, disease stage, and survival remains unclear. Additionally, we did not account for treatment delay which was reported to have independently increased mortality risk [40]. Furthermore, ACCI and ACE-27 as indicators of comorbidity, Clavien-Dindo classification system to assess surgical complications and preoperative thrombocyte count were only collected for early and late mortality group patients. Lastly, during the time frame between 2012 and 2015, the 7th edition of the UICC TNM staging system was used in Finland. The UICC 8th edition introduces updates, particularly regarding HPV-associated oropharyngeal cancer, which may influence staging accuracy. However, applying the UICC 8th edition retrospectively could lead to inconsistencies in data interpretation, as treatment decisions and documentation were based on the earlier system.

The main strength of this study is that it can be regarded as a population-based series as practically all HNSCC patients of the Helsinki University Hospital referral area were included during the study period. Finland has a population of approximately 5.6 million inhabitants [41], and the HUS catchment area encompasses nearly 2.2 million inhabitants [42]. According to the Finnish Cancer Registry, the number of new annual HNC cases is relative to the population size within each hospital’s catchment area. Also, according to the Finnish Cancer Registry’s statistics in 2022, the Southern Finland collaborative area accounted for approximately 40% of the total oropharyngeal and tongue cancer cases in Finland during 2018–2022. However, there may be some dropouts, as only since 2018, the treatment planning of HNC by a multidisciplinary team has been centralized to the five university hospitals of Finland by law. Consequently, some patients diagnosed with small tumors may have received treatment at district hospitals during the specified time frame. Besides the relatively large cohort size, further strengths of our study lie in the absence of selection bias caused by socioeconomic or insurance status-related issues, given its population-based nature and the fact that all treatments within the HUS referral area are administered through our hospital, ensuring the homogeneity of healthcare provided to all patients.

## Conclusions

The death rate during or within 6 months after treatment with curative intent was 10.1%. Advanced T and N class, over 40 pack-years of smoking, and heavy alcohol consumption were associated with increased odds of early death. Yet, only age, along with T4, N2, and N3 class were independent risk factors for early death. Additionally, elevated thrombocyte levels >380 (×10^9^L) were observed in the early mortality group. As previously known, older patients with advanced disease have the highest risk of early death. Further prospective research on thrombocyte count as a readily measurable prognostic marker is required to assess its role in identifying patients at risk of early death. This could help clinicians assess their patients’ risk of early death and to find effective strategies to mitigate it.

## Data Availability

Data is available upon request from the corresponding author.
